# SARS-CoV-2 infection of the central nervous system in a 14-month-old child: A case report of a complete autopsy

**DOI:** 10.1016/j.lana.2021.100046

**Published:** 2021-08-28

**Authors:** Ismael Gomes, Karina Karmirian, Júlia T. Oliveira, Carolina da S.G. Pedrosa, Mayara Abud Mendes, Fernando Colonna Rosman, Leila Chimelli, Stevens Rehen

**Affiliations:** 1 Anatomic Pathology Service, Jesus Municipal Hospital, Rio de Janeiro, RJ, Brazil; 2 Department of Genetics, Institute of Biology, Federal University of Rio de Janeiro (UFRJ), Rio de Janeiro, RJ, Brazil; 3 D'Or Institute for Research and Education (IDOR), Rio de Janeiro, RJ, Brazil; 4 Institute of Biomedical Sciences, Federal University of Rio de Janeiro (UFRJ), Rio de Janeiro, RJ, Brazil; 5 Laboratory of Neuropathology, State Institute of Brain Paulo Niemeyer, Post-Graduate Programs of Pathology and Translational Neuroscience, UFRJ, Rio de Janeiro, RJ, Brazil; 6 Department of Pathology, Faculty of Medicine, UFRJ, Rio de Janeiro, RJ, Brazil

**Keywords:** SARS-CoV-2, neuroinvasion, choroid plexus, COVID-19, child, autopsy

## Abstract

**Background:**

Neurological and other systemic complications occur in adults with severe COVID-19. Here we describe SARS-CoV-2 infection complicated by neuroinvasion in the *post-mortem* tissues of a child.

**Methods:**

We performed a complete autopsy of a 14-month-old child who died of COVID-19 pneumonitis. Histological sections of multiple organs were stained with haematoxylin and eosin. Luxol fast blue staining for myelin and immunohistochemistry were performed in selected areas of the brain. The presence of SARS-CoV-2 was investigated by immunostaining with anti-spike protein antibody and by RT-qPCR.

**Findings:**

Lesions included microthrombosis, pulmonary congestion, interstitial oedema, lymphocytic infiltrates, bronchiolar injury, collapsed alveolar spaces, cortical atrophy, and severe neuronal loss. SARS-CoV-2 staining was observed along the apical region of the choroid plexus (ChP) epithelium and in ependymal cells of the lateral ventricle, but was restricted to ChP capillaries and vessels in some regions. SARS-CoV-2 infection of brain tissue was confirmed by RT-qPCR in fragments of the ChP, lateral ventricle, and cortex.

**Interpretation:**

Our results show multisystemic histopathological alterations caused by SARS-CoV-2 infection and contribute to knowledge regarding the course of fatal COVID-19 in children. Furthermore, our findings of ChP infection and viral neurotropism suggest that SARS-CoV-2 may invade the central nervous system by blood-cerebrospinal fluid barrier disruption.

**Funding:**

Carlos Chagas Filho Foundation for Supporting Research in the State of Rio de Janeiro (FAPERJ); the National Council for Scientific and Technological Development (CNPq) and Coordination for the Improvement of Higher Education Personnel (CAPES), in addition to intramural grants from D'Or Institute for Research and Education.

**Editor's Note:**

This translation in Portuguese was submitted by the authors and we reproduce it as supplied. It has not been peer reviewed. Our editorial processes have only been applied to the original abstract in English, which should serve as reference for this manuscript.

**Resumo:**

Complicações sistêmicas e neurológicas foram descritas em adultos com COVID-19 grave. Neste trabalho, descrevemos a infecção por SARS-CoV-2, incluindo sua neuroinvasão, nos tecidos *post-mortem* de uma criança.

**Métodos:**

Realizamos a autópsia completa de uma criança de 14 meses que morreu de pneumonite por COVID-19. Cortes histológicos de múltiplos órgãos foram corados com Hematoxilina e Eosina. A coloração de Luxol Fast Blue para mielina e imuno-histoquímica foram realizadas em áreas selecionadas do cérebro. A presença de SARS-CoV-2 foi investigada por imunomarcação com anticorpo anti-proteína spike e por RT-qPCR.

**Achados:**

As lesões incluíram microtrombose, congestão pulmonar, edema intersticial, infiltrados linfocíticos, lesão bronquiolar, colapso dos espaços alveolares, atrofia cortical e perda neuronal grave. A presença de SARS-CoV-2 foi observada ao longo da região apical do epitélio do plexo coróide (PC) e nas células ependimárias do ventrículo lateral, mas ficou restrita aos capilares e vasos do PC em outras regiões. A infecção do tecido cerebral por SARS-CoV-2 foi confirmada por RT-qPCR em fragmentos do PC, ventrículo lateral e cortex cerebral.

**Interpretação:**

Nossos resultados mostram alterações histopatológicas multissistêmicas causadas pela infecção por SARS-CoV-2 e contribuem para ampliar o conhecimento sobre a evolução da COVID-19 fatal em crianças. Além disso, nossos achados sobre a infecção no PC e neurotropismo viral sugerem que o SARS-CoV-2 pode invadir o sistema nervoso central pela ruptura da barreira sangue-líquido cefalorraquidiano.

**Financiamento:**

Fundação de Amparo à Pesquisa do Estado do Rio de Janeiro (FAPERJ); Conselho Nacional de Desenvolvimento Científico e Tecnológico (CNPQ) e Coordenação de Aperfeiçoamento de Pessoal de Nível Superior (CAPES), além de financiamento intramural do Instituto D'Or de Pesquisa e Educação.

**Research in context**

**Evidence before this study**

We searched PubMed and bioRxiv databases from August 1, 2020, until July 7, 2021, without language restrictions. Search terms included: ``SARS-CoV-2,'' ``COVID-19,'' ``central nervous system,'' ``neuroinvasion,'' and ``choroid plexus.'' We focused on publications describing SARS-COV-2 infection of the central nervous system (CNS) as well as neurological complications of COVID-19. We also searched for in vitro and in vivo studies that investigated the route of SARS-CoV-2 entry into the CNS.

**Added value of this study**

This study adds information regarding the histopathological findings of fatal COVID-19 in a 14-month-old child, and suggests a role of choroid plexus infection as the portal of viral entry into the CNS. Immunofluorescence and RT-qPCR analyses disclosed SARS-CoV-2 infection of the cerebral cortex, lateral ventricle, cerebellum, basal ganglia, and choroid plexus. Additionally, intense SARS-CoV-2 staining was observed along the choroid plexus endothelium, suggesting a role of blood-CSF barrier disruption in neuroinvasion. These data are valuable for understanding the neuroinvasive profile of SARS-CoV-2, and corroborate previous results addressing choroid plexus infection. Furthermore, this study provides multisystemic histopathological findings from a complete autopsy of a child who died of fulminant COVID-19. In addition to coronavirus pneumonitis, microthrombosis was observed in lung, thyroid, and myocardium. Multiple organ damage included ischemic necrosis of the pancreas, hepatic steatosis, laryngitis, sialadenitis, esophagitis, and diffuse osmotic nephrosis. Neuropathological analysis revealed extensive atrophy, cortical laminar necrosis, and reactive gliosis, although the extent of CNS injury could not be attributed exclusively to SARS-CoV-2 infection. Despite the patient’s chronic seizure disorder, the complete autopsy performed in this study, together with the characterization of SARS-CoV-2 infection of the CNS, add relevant data on the systemic and neurological manifestations of COVID-19.

**Implications of all the available evidence**

This case report supports the hypothesis that SARS-CoV-2 neuroinvasion may result from hematogenous seeding of the choroid plexus followed by blood-CSF barrier disruption. However, further studies are needed to elucidate the role of choroid plexus infection in CNS pathogenesis. Together with previous reports, our data emphasize the need for additional investigations of severe and fatal cases of COVID-19 in children - even at lower incidence rates - to better understand the systemic manifestations of COVID-19 and to inform therapeutic strategies.

Alt-text: Unlabelled box

## 1 Introduction

COVID-19, caused by Severe Acute Respiratory Syndrome Coronavirus-2 (SARS-CoV-2), emerged in Wuhan, China in December 2019, and spread rapidly across the globe, causing approximately 4 million deaths by early July 2021 [1]. Initially characterized as a respiratory illness, COVID-19 is now described as a multisystemic infectious disease with significant complications in multiple organs [2].

Major complications include neurological manifestations that occur in up to 67% of severely affected patients [3]. Neurological sequelae may be acute or chronic, and may include headache, vomiting, dizziness, hypogeusia and hyposmia, persistent fatigue, memory dysfunction, gait disorders, and meningitis/encephalitis [4], [5], [6]. A *post-mortem* case series detected SARS-CoV-2 in the brains of 53% of patients who died of COVID-19, with viral proteins in cranial nerves and in isolated cells of the brainstem [7]. As suggested previously for SARS-CoV, neuroinvasion of the brainstem cardiorespiratory centre may promote respiratory failure in COVID-19 [8]. The SARS-CoV-2 spike protein has been demonstrated in cortical neurons and in cerebrovascular endothelium [9]. Although detection of SARS-CoV-2 RNA in cerebrospinal fluid (CSF) is uncommon, it has been reported in two adults [10] and one infant [11].

The first proposed neuroinvasive pathway of SARS-CoV-2 was anterograde axonal transport via olfactory neurons. A recent study detected SARS-CoV-2 virions in nerve endings of olfactory mucosae [12], whereas a rodent study demonstrated that SARS-CoV-2 primarily infects non-neural cells of the olfactory epithelium [13]. Furthermore, central nervous system (CNS) invasion with infection of cortical neurons has also been described [9]. Viral-like particles have been demonstrated in cerebrovascular endothelial cells in *post-mortem* brain tissue of COVID-19 patients, suggesting hematogenous dissemination as another route to the CNS [9]. The brain parenchyma is separated from the systemic circulation by the blood-brain barrier, a complex and highly insulating barrier formed primarily by endothelial cells attached by tight junctions [14]. In contrast, the blood-CSF barrier is comprised of a single layer of choroid plexus (ChP) epithelial cells that separates the CSF from fenestrated stromal capillaries [15], acting as a gateway for immune cells to enter the brain [16]. This intimate interaction between blood, immune cells, and the ChP epithelium is a source of CSF vulnerability. In view of these conflicting findings, the mechanisms of SARS-CoV-2 entry into the CNS remain a point of debate.

Here we describe multi-organ pathological alterations in the *post-mortem* tissues of a 14-month-old child, who died of respiratory failure that complicated COVID-19. Our findings revealed microthrombi in veins and arteries in addition to pulmonary involvement resulting in congestion, bronchiolar injury, and collapsed alveolar spaces. The brain exhibited severe atrophy and neuronal loss. SARS-CoV-2 spike protein (SP) was demonstrated by immunostaining along the ChP epithelium and ependymal cells of the lateral ventricle, and in ChP capillaries and vessels. Furthermore, RT-qPCR for genes encoding nucleocapsid proteins confirmed the presence of SARS-CoV-2 in fragments of ChP, lateral ventricle, and cortex. Our findings endorse literature that indicate that SARS-CoV-2 infection of the ChP may disrupt the blood-CSF barrier, thereby potentiating CNS invasion and pathogenesis.

## 2 Methods

### 2.1 Patient data

A black 14-month-old female child who died of respiratory failure caused by bilateral COVID-19 pneumonitis and massive pancreatic ischemic necrosis secondary to a large vessel thrombosis.

### 2.2 Autopsy procedures

A full autopsy was performed with a *post-mortem* interval of 37 hours, following best practices autopsy guidelines according to biosafety practices in Anatomical Pathology Laboratories. All tissues were fixed with 10% buffered formalin (pH 7.4) for at least 48 hours and further processed using a standard protocol for paraffin embedding.

### 2.3 Immunofluorescence

Paraffin blocks from lungs and brain (ChP, cerebral cortex, globus pallidus, lateral ventricle, medulla oblongata, midbrain, pons, and putamen) were selected to produce a tissue microarray, as adapted from Pires et al. [17]. Four-µm sections were deparaffinized and rehydrated, subjected to antigen retrieval using 10 mM citrate buffer (pH 6.0) for 30 min at 98°C, and were then blocked/permeabilized (3% bovine serum albumin/0.3% Triton X-100) for 1 h. Overnight incubation at 4°C was performed with anti-SARS-CoV-2 SP monoclonal antibody (Genetex, Cat GTX632604, 1:500); convalescent serum (CS, 1:1000) or anti-dsRNA (J2) (Merck-Millipore Cat MABE1134, 1:200). The slides were then washed with PBS and incubated with secondary antibody (Goat anti-Mouse Alexa Fluor 488, 1:400; A-11001) for 45 min at 37°C. Nuclei were stained with 0.5 µg/mL 4′-6-diamino-2-phenylindole for 5 minutes, and the slides were mounted with Aqua-Poly-mount (Polysciences, Pennsylvania, USA). Images were acquired with a Leica TCS SP8 confocal microscope using a 63x/oil objective lens.

### 2.4 Immunohistochemistry

Immunohistochemical reactions were performed in selected areas of nervous tissue which presented histological lesions, using the following monoclonal antibodies (Cell Marque, Sigma-Aldrich Co, Rocklin, CA, USA) and dilutions: anti-glial fibrillary acidic protein- GFAP- clone EP672y (1:500), anti-NeuN - Clone A100 (Zeta Corporation, Arcadia, CA, USA) (1:200), CD3 - Clone MRQ-39 (1:1000), CD20 - Clone SP-32 (1:1000), and CD68 - Clone Kp-1 (1:1000), according to standard protocols [18]. Five-µm-thick tissue sections were incubated in a drying oven at 37°C for 6 h and then deparaffinized in xylene. Tissue sections were rehydrated by placement in decreasing concentrations of alcohol, followed by washing in distilled water. To enhance antigen retrieval, the tissue sections were pre-treated in an electric pressure cooker for 15 min in a solution of 1:20 Declere® (pH 6)/1:100 Trilogy (pH 9) in distilled water. To block endogenous peroxidase activity, tissue sections were exposed to hydrogen peroxide, washed with distilled water, and rinsed in phosphate buffered saline (PBS) to stop enzymatic digestion. They were then incubated with the primary antibody overnight at 4°C, rinsed in PBS for 5 min, and incubated with Polymer Hi Def (horseradish peroxidase system) for 10 min at room temperature preceded by several washes in PBS. The peroxidase reaction was visualized with DAB substrate rinsed in running water; the sections were then counterstained with Meyer's haematoxylin for 1 min, washed in running tap water for 3 min, dehydrated in alcohol, cleared in xylene, and mounted in resinous medium. Images were acquired with an Axio Carl Zeiss Scop A1 microscope using 4x, 10x, 20x, and 40x objective lenses.

### 2.5 Total RNA isolation from human specimens

Tissue samples from heart, brain (frontal cortex, central core, lateral ventricle, ChP, cerebellum); trachea; larynx; kidney; liver; stomach; and lung were sliced into thick sections. Subsequently, specimens were transferred to 3.0 mm TriplePure™ Zirconium homogenizer beads (Benchmark Scientific, New Jersey, USA) and shaken vigorously using the BeadBug^TM^ Microtube Homogenizer apparatus (D1030-E, Benchmark Scientific). Total RNA was isolated in 1 mL of TRIzol™ Reagent (Thermo Fisher Scientific, Massachusetts, USA), according to the manufacturer's instructions. Additionally, total RNA was isolated from 140 μL of upper respiratory tract specimens obtained from nasopharyngeal swabs from four adult subjects using the QIAamp® Viral RNA Mini Kit (52906, Qiagen, Hilden, Germany), following the manufacturer's instructions. RNA was eluted in 60 µL. Total RNA from human induced pluripotent stem cell (iPSC)-derived astrocytes was extracted using the PureLink™ RNA Mini Kit (12183018A, Thermo Fisher Scientific), according to the manufacturer's protocol.

### 2.6 Detection of SARS-CoV-2 RNA in human specimens by RT-qPCR

RT–qPCR was performed on each sample using the 2019–nCoV CDC RUO Kit (IDT: 10006713) and 2019–nCoV CDC RUO Primers and Probes (PN: 10006713) for the detection of viral RNA (SARS-CoV-2 nucleocapsid N1- and N2-encoding fragments) and the RNase P (RP) primer set for the detection of human RNase P RNA (Integrated DNA Technologies, Iowa, USA). For each specimen, three separated reactions were set up in a 96-well plate including N1, N2, and RP primers and probes. RT-qPCR was conducted with a total reaction volume of 20 µL containing 15 µL of GoTaq® Probe 1-Step RT-qPCR System (A6120, Promega, Wisconsin, USA) comprised of the following components: 3.1 µL ultrapure water, 10 µL GoTaq® Probe qPCR Master Mix with dUTP (2X), 0.4 µL GoScriptTM RT Mix for 1-Step RT-qPCR, 1.5 µL primer/probe sets for either *N1, N2*, or *RP* (IDT) and 5 µL of extracted RNA. To monitor assay performance, all reactions were conducted with negative controls (human specimen controls – human iPSC-derived astrocytes; paediatric *post-mortem* lung tissue of a four-month-old infant who died of respiratory failure caused by bilateral non-COVID pneumonitis; adult nasopharyngeal swabs - all negative for respiratory viruses – and no template control - with UltraPure DNase/RNase-Free Distilled water instead of RNA template); and positive controls (2019-nCoV_N Positive Control plasmid, IDT: 10006625; Hs_RPP30 (human ribonuclease P/MRP subunit p30) Positive Control, IDT: 10006626) and adult nasopharyngeal swabs positive for COVID-19), which were incorporated into each run to ensure proper testing controls. Briefly, reactions were performed on a StepOnePlus™ Real-Time PCR System thermocycler (Thermo Fisher Scientific). Thermal cycling conditions comprised a holding stage at 45°C for 15 min, 95°C for 2 min, followed by 45 cycles of denaturation at 95°C for 3 sec, and annealing and extension at 55°C for 30 sec. RT-qPCR results were analysed according to “FDA Inform Diagnostics SARS-CoV-2 RT-PCR Assay”, available at https://www.fda.gov/media/139572/download.

### 2.7 Standard Curve and Data Analysis

The standard curve was prepared with 10-fold serial dilutions, ranging from 1 × 10^6^ to 10 copies/reaction of synthetic viral RNA in a human matrix containing pooled RNA isolated from four contrived human respiratory specimens - all negative for respiratory viruses - obtained from nasopharyngeal swabs. Each dilution point was run in triplicate.

Data analysis started with threshold adjustments for individual targets that minimized huge discrepancies using Applied Biosystems StepOnePlus™ software v.4.3. Then, crossing points (Cp) were exported and imported into R software and environment for statistical computing v.3.6.3. For standard curve fitting, both an ordinary least squares (OLS) linear model and a robust linear model (M estimator) were fitted. Because the difference was negligible, the OLS linear model was used to fit a line and calculate estimates for intercept and slopes (log10 scale). RT-qPCR efficiency was calculated using E = 10(-1/slope). Cps from RT-qPCR replicates were used individually as inputs to calculate quantity (using stats::predict.lm() function) and then transformed by the antilog. Copy numbers were summarized per sample with the arithmetic mean and sample standard deviation. Scatter plots with best fitted lines were drawn with ggplot2 v.3.3.0.

### 2.8 Ethical Statement

The research protocol was approved by the local Ethics Committee (Copa D'Or Hospital/Instituto D'Or de Pesquisa e Ensino, IDOR, CAAE number: 37211220.0.0000.5249), and was performed according to the Declaration of Helsinki. The use of the convalescent serum from COVID-19 patients was approved by CAAE number: 30650420.4.1001.0008.

### 2.9 Role of the funding source

The funders of the study had no role in the study design, data collection, data analysis, interpretation, or writing of the report.

## 3 Results

### 3.1 Clinical History

The child was born at term, and experienced normal general health and neurological development. At 10 months of age, she was admitted to an emergency hospital due to vomiting, hypotonia, and episodic seizures. Viral meningitis was suspected, although no specific laboratory testing was performed to confirm the diagnosis. After 16 days of hospitalization, she was discharged with normal and stable vital signs. In the succeeding months, she was hospitalized three times with similar symptoms of sporadic seizures, hypotonia, and vomiting. During the third re-admission, her electroencephalogram was abnormal with diffuse slow activity. CSF analysis revealed normal levels of protein (63 mg/dL) and glucose (62 mg/dL), non-reactive VDRL, and negative Gram's stain and bacterial culture. She was discharged from hospital with a prescription for phenobarbital (5 mg/Kg/day). Thirty days after the last hospitalization, she was admitted to a paediatric hospital, presenting with vomiting and repetitive movements of the left upper and lower extremities, hypotonia, postural instability, and inability to support her head, sit, and walk. Pulmonary auscultation revealed a universally audible vesicular murmur, wheezing, rhonchi, and rales. The oxygen saturation was low to normal (92-98% SpO2). Eight days post-admission, cranial computed tomography showed abnormal enlargement of the third and lateral ventricles suggesting central white matter volume loss and/or relative obstruction of CSF flow at the level of the cerebral aqueduct. Moreover, there was loss of grey-white differentiation consistent with diffuse ischemic injury (Supplementary Figure 1). However, she was conscious, active, and afebrile, presenting stable clinical features, such as normal respiratory rate (24 breaths per minute) and 97-100% SpO2 with non-invasive ventilation. Fifteen days post-admission, her illness progressed. Due to suspicion of pneumonia, tracheal secretions were obtained and tested positive for SARS-CoV-2 by RT-qPCR. Pneumonia worsened acutely, requiring supplemental oxygenation and invasive mechanical ventilation. She remained hemodynamically stable but developed metabolic acidosis, respiratory alkalosis, and impaired consciousness (Glasgow scale 3). During the final days of hospitalization, she was hemodynamically unstable, progressing to severe hypoxemia and death due to respiratory failure. The laboratory parameters evaluated during her hospitalization are listed in Table 1, and the prescribed medications are listed in Supplementary Table 1.

**Table 1:** Patient's laboratory parameters at different post-admission timepoints

	Days post-admission						
Laboratory parameters	4	10	14	17	23	25	Normal range
**Haemoglobin, g/dL**	16.6	12.3	12.3	9.9	10.1	10.6	12.6 ± 1.5
**Hematocrit, %**	48.5	35.6	35.9	..	29.6	32	34 ± 4
**Platelets, × 10^3 cells/μL**	304	..	343	295	383	393	200 - 550
**Erythrocytes, x10^6/uL**	5.7	4.25	..	..	..	..	4.04 - 6.13
**Leukocytes, × 10^3 cells/μL**	15.7	20.2	32.7	17.5	24.6	35.8	6 - 16
**Lymphocytes, %**	41	18	10	16	20	13	10 - 50
**Segmented neutrophils, %**	43	48	73	75	67	58	37 - 80
**Band neutrophils, U/μL**	3	1	4	2	7	25	300
**Alkaline phosphatase, U/L**	..	1171	..	928	..	1086	65 - 645
**Creatinine, mg/dL**	0.4	0.4	0.3	0.3	0.3	0.3	0.3 – 0.8
**Urea, mg/dL**	25	14	27	22	13	17	10 - 50
**C-reactive protein, mg/L**	2	0.8	6.1	26.7	108.1	..	0 - 5
**Aspartate aminotransferase, U/L**	131	78	69	175	106	114	0 - 38
**Alanine aminotransferase, U/L**	47	71	82	197	100	129	0 - 42
**Total bilirubin, mg/dL**	..	0.11	..	..	..	0.18	0.2 - 1

### 3.2 Autopsy Findings

**Figure 1:**
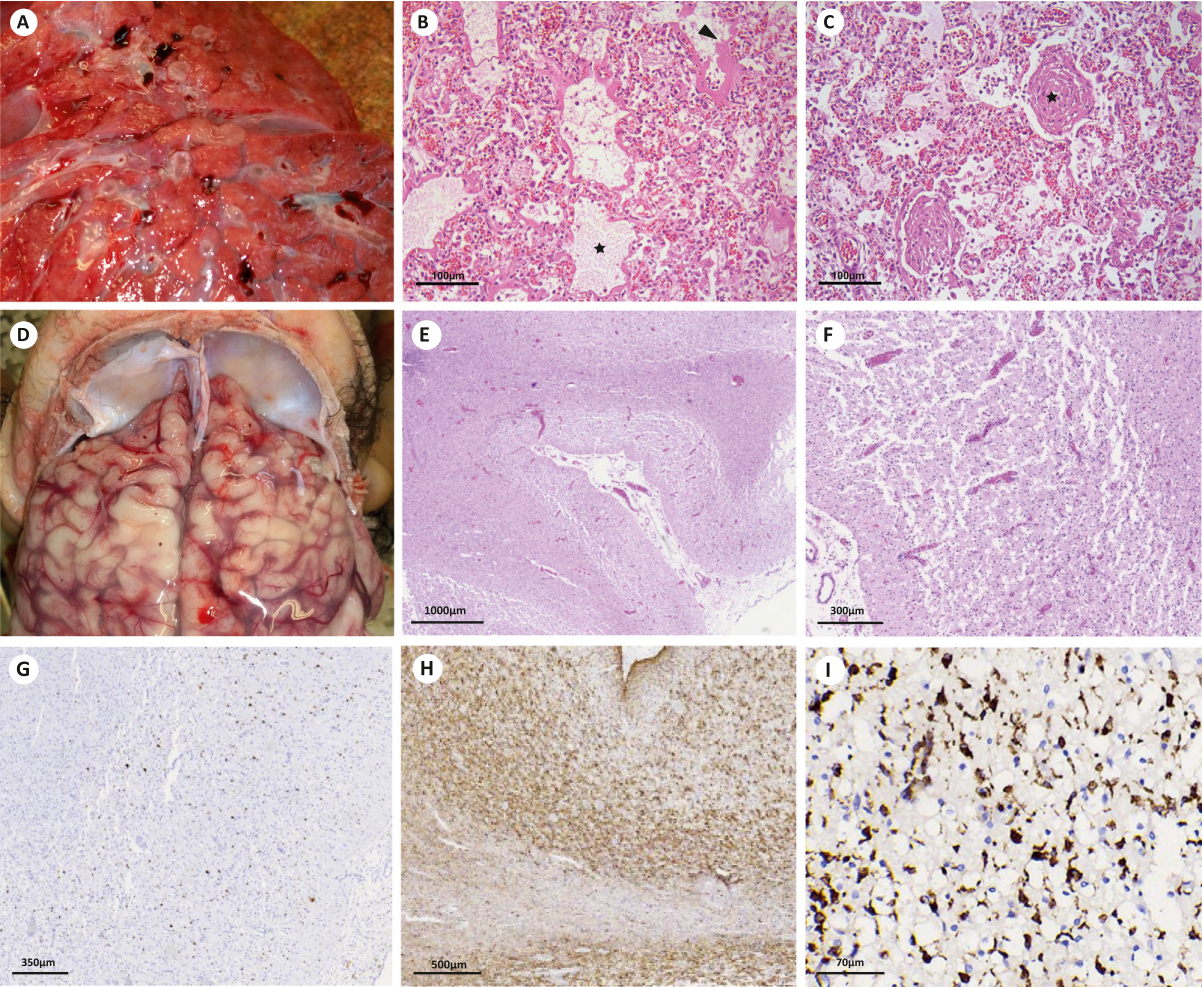
**Macroscopic and microscopic appearance of pulmonary and brain tissues. (A)** Cut surface of congested and oedematous lungs. The pulmonary acini (lobules) are well delimited due to pneumonitis. *Post-mortem* clots are seen in branches of pulmonary arteries. **(B)** Diffuse damage of respiratory bronchioles (star) associated with eosinophilic hyaline membranes (arrowhead). Note the congestion of alveolar capillaries, the collapsed alveolar spaces, and the lymphocytic interstitial infiltrate. Scale bar: 100 µm. **(C)** Respiratory bronchioles with eosinophilic plugs (star) of plasma proteins and cellular debris. Observe the collapsed alveolar spaces and hyaline membranes. Scale bar: 100 µm. **(D)** Unfixed brain in situ does not fill the base of the skull due to marked atrophy. **(E)** Histological section of cortex and white matter, showing cortical laminar necrosis. Scale bar: 1000 µm. Detail in **(F)** Scale bar: 300 µm. There is severe nerve cell loss and vascular proliferation, but the molecular layer (left) is relatively spared. **(G)** Immunostaining for NeuN demonstrating severe cortical neuronal loss. Scale bar: 350 µm. **(H)** Immunostaining for GFAP revealing severe reactive gliosis involving cortex and white matter. Scale bar: 500 µm. **(I)** Immunostaining for CD68 exhibiting microglial and macrophage proliferation in cortex and white matter. Scale bar: 70 µm.

A complete autopsy revealed damage to multiple organs, including venous and arterial microthrombosis in several regions (Supplementary Figure 2). A complete report of the morphological findings is provided in Table 2 and Supplementary Figure 3. As expected, COVID-19 pneumonitis caused lung congestion and oedema. Interstitial inflammation delineated the pulmonary acini (Figure 1A). Histopathological findings of the lung were characterized by bronchial lymphoid aggregates, interstitial lymphocytic infiltrates, and diffuse bronchiolar damage covered by hyaline membranes, along with collapsed alveolar spaces, some of which contained eosinophilic plugs of plasma proteins and cellular debris (Figure 1B, C). In addition, we found congestion, oedema, haemorrhagic foci, atelectasis, and recent microthrombi in pulmonary artery branches of both lungs (Supplementary Figure 2A).

**Table 2:** Morphological findings in child *post-mortem* tissue samples.

Organs	Macroscopic Examination	Histopathologic findings
Brain	Marked atrophy, edema, hydrocephalus ex vacuo.	Atrophic cerebral cortex, laminar/massive cortical neuronal loss, spongiosis, gliosis, microgliosis, and macrophages. Diffuse white matter edema, focal perivascular, and neuronal mineralization. Mild focal lymphocytic infiltrate in the leptomeninges.
Heart	Normal	Microthrombi in small arteries of the left ventricle. Focal mild lymphocytic infiltrate in the right ventricle epicardium.
Larynx	..	Laryngitis: moderate lymphocytic inflammatory infiltrate in the mucosa and the submucosa, associated or not with mucosal erosion, necrosis, and fibrinonecrotic membrane.
Trachea	..	Mild lymphocytic infiltrate in some regions of the mucosa and submucosa. Focal squamous metaplasia.
Lungs	Enlarged, with congestion, edema and well demarcated lobules. Pleural effusion.	Pneumonitis: diffuse respiratory bronchiolar damage with hyaline membranes and collapsed alveolar spaces, associated with interstitial lymphocytic inflammation, plugs of plasma proteins and cellular debris in bronchiolar lumina, occasionally with macrophages, and lymphoid aggregates in their walls. Pneumocyte type II proliferation in distal respiratory spaces. Congestion, edema, and some foci of hemorrhage and atelectasis. Microthrombi in some small pulmonary arteries. Pleura with mild lymphocytic infiltrate, congestion, and edema.
Submandibular salivary glands	..	Sialadenitis: focal moderate lymphocytic infiltration in both salivary glands.
Tongue	..	Severe lymphoid hypoplasia in the posterior region and mild interstitial lymphocytic infiltrate around some small salivary glands.
Esophagus	..	Esophagitis: focal lymphocytic infiltration of the mucosa, along with lymphocyte exocytosis. Regional venular thrombosis.
Stomach	..	Mild gastritis, some lymphoid aggregates, congestion and foci of superficial hemorrhage of the mucosa.
Intestines	..	Lymphoid aggregates in the mucosa and submucosa.
Liver	Pale red	Steatosis. Mild lymphocytic infiltration in some portal spaces; occasional recent venular microthrombi.
Pancreas	Dark gray with black areas	Ischemic necrosis of the head, body and tail of the pancreas with hemorrhage. Ischemic steatonecrosis of the regional adipose tissue. Ischemic necrosis of the regional lymph nodes. There was no pancreatitis.
Kidneys	..	Diffuse osmotic nephrosis secondary to hydroelectrolytic disorders. Some microthrombi in small arteries.
Thymus	..	Severe diffuse lymphoid hypoplasia.
Vermiform appendix	..	Severe diffuse lymphoid hypoplasia.
Lymph nodes	..	Moderate lymphoid hypoplasia.
Spleen	..	White pulp lacking germinal centers.
Thyroid	..	Small follicles. Microthrombi in regional small arteries and veins.
Retroperitoneal striated muscle	..	Focal myositis with lymphocytic inflammatory infiltrate.
Pelvic vein	..	Focal thrombophlebitis associated with apoptosis of leukocytes in the wall.

A comprehensive analysis of the brain showed severe cerebral atrophy with a large remaining space within the cranial cavity (Figure 1D). The brain weight (635 grams) was about 33% less than normal for age (range weight: 940 to 1,010 grams). Indeed, we observed significant atrophy in sections of the cerebral hemispheres and thinner, granulated, and discoloured cortical surfaces (Supplementary Figure 4A). There were focal cortical depressions corresponding to areas of cortical collapse, particularly in the temporal lobes, which were extremely soft. The cerebellum appeared normal but the basis pontis was smaller than the expected average for the patient's age. The olfactory nerves and bulbs were not preserved because of cerebral softening.

We also observed severe tissue injury in many regions of the cerebral cortex that displayed laminar cortical necrosis, spongiosis, microvascular proliferation, and diffuse cerebral oedema (Figure 1E, F). Pan-necrotic cortical lesions featured profound neuronal loss, particularly in the temporal lobes. Far-advanced loss of cortical neurons was highlighted with immunostaining for NeuN (Figure 1G). Additionally, we observed neuronal loss in the basal ganglia and thalamus, and also noticed severe reactive gliosis involving basal ganglia and the periventricular region (Figure 1H) and microglial/macrophage proliferation (figure 1I). White matter was preserved (Supplementary Figure 4B). The cerebellum was relatively preserved with occasional red or shrunken Purkinje cells, which were scarce in some regions. The brainstem presented a few descending axons.

**Figure 2:**
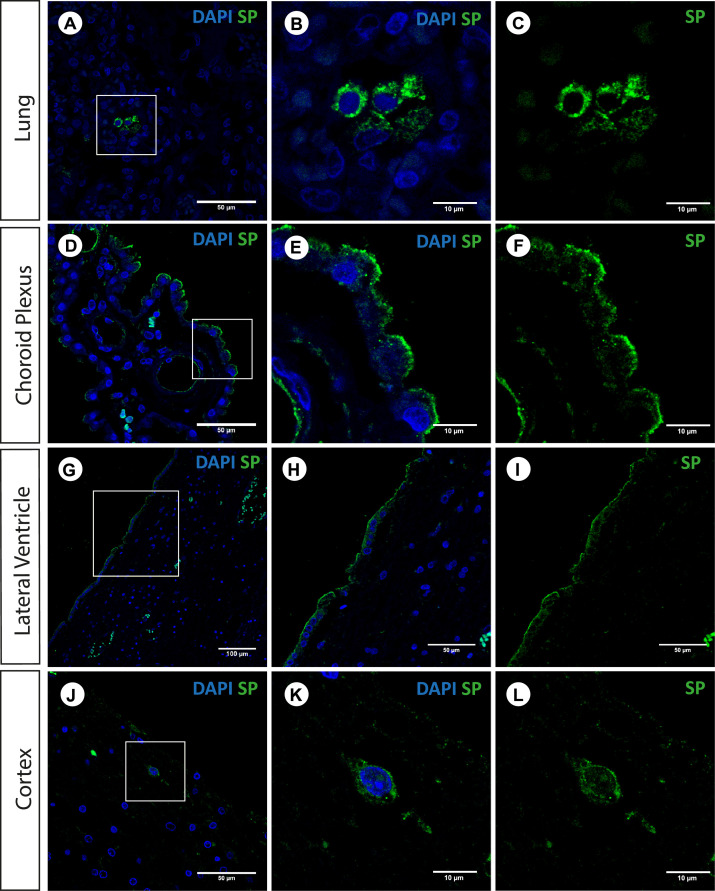
**SARS-CoV-2 detection in lungs, choroid plexus, lateral ventricle and cerebral cortex.** Photomicrographs of immunostaining for spike protein in lung tissue **(A-C)** and in the brain (choroid plexus - **D-F**; lateral ventricle - **G-I**; cerebral cortex - **J-L**). Scale bars: (A, D, H, I, J) 50 µm; (G) 100 µm; (B, C, E, F, K, L) 10 µm.

Immunostaining to detect the presence of SARS-CoV-2 SP in lung tissue yielded positive results in groups of adjacent pulmonary parenchymal cells, as expected (Figure 2A-C). We observed intense SARS-CoV-2 staining in the brain along the apical region of the ChP epithelium (Figure 2D-F). We also detected SARS-CoV-2 positive cells, to a lesser extent, in the ependyma of the lateral ventricle (Figure 2G-I); considering that the ChP extends within each cerebral ventricle, this finding was unsurprising. Furthermore, we observed scarce spike protein-positive cells in the cortex (Figure 2J-L), and no positive cells in the medulla oblongata, pons, midbrain, and putamen, nor in control samples from non-COVID-19 patients (Supplementary Figure 5). SARS-CoV-2 infection was further confirmed by the detection of nucleocapsid genes *N1* and *N2* by RT-qPCR in distinct fragments from the lung, choroid plexus, lateral ventricle, and cortex (Table 3). The presence of elevated numbers of viral RNA copies per reaction in representative fragments of cortex and lung when compared with other analysed tissues was notable. We also detected *N1* and *N2* genes in fragments from a wide variety of other tissue samples such as basal ganglia, cerebellum, heart, kidney, liver, stomach, trachea, and larynx (Supplementary Table 2).

**Table 3:** 2019-nCoV RTq-PCR diagnostic panel results of child *post-mortem* tissue samples

Specimen Type	2019-nCoV_N1 Assay	2019-nCoV_N2 Assay	Hs_RPP30	Result Interpretation
	(mean copies ± SD)	(mean copies ± SD)	(Cp mean ± SD)	(Detected/Total Tested)
**Lung**	2,408 (±3,276)	1,316 (±676)	34.5 (± 2.7)	2019-nCoV detected (3/3)
**Choroid Plexus**	Undetermined	30	32.4	2019-nCoV detected (1/1)
**Lateral Ventricle**	4	23 (±12)	35.0 (± 1.2)	2019-nCoV detected (1/3)
**Cortex**	684 (±680)	2,996 (±3,714)	27.8 (± 1.2)	2019-nCoV detected (2/2)
**Human Specimen Control** (Child lung tissue - Negative Control)	ND	ND	24.3	2019-nCoV not detected (2/2)

RT-qPCR detection of SARS-CoV-2 regions of nucleocapsid (N) genes (N1 and N2). The mean and standard deviations of absolute quantification (number of copies/reactions) of N1 and N2, and mean and standard deviations of crossing points (Cp) values of RPP30 were calculated from data obtained in all analysed distinct fragments from the same *post-mortem* tissue specimens of lung (n = 3), choroid plexus (n = 1), lateral ventricle (n = 3), and cortex (n = 2). The complete panel of SARS-CoV-2 detection in child tissue samples is demonstrated in **Supplementary Table 2**. RNA isolated from a *post-mortem* lung sample derived from a non-Covid-19 child was used as a negative control. RP: human RNase P gene; ND, not determined.

**Figure 3:**
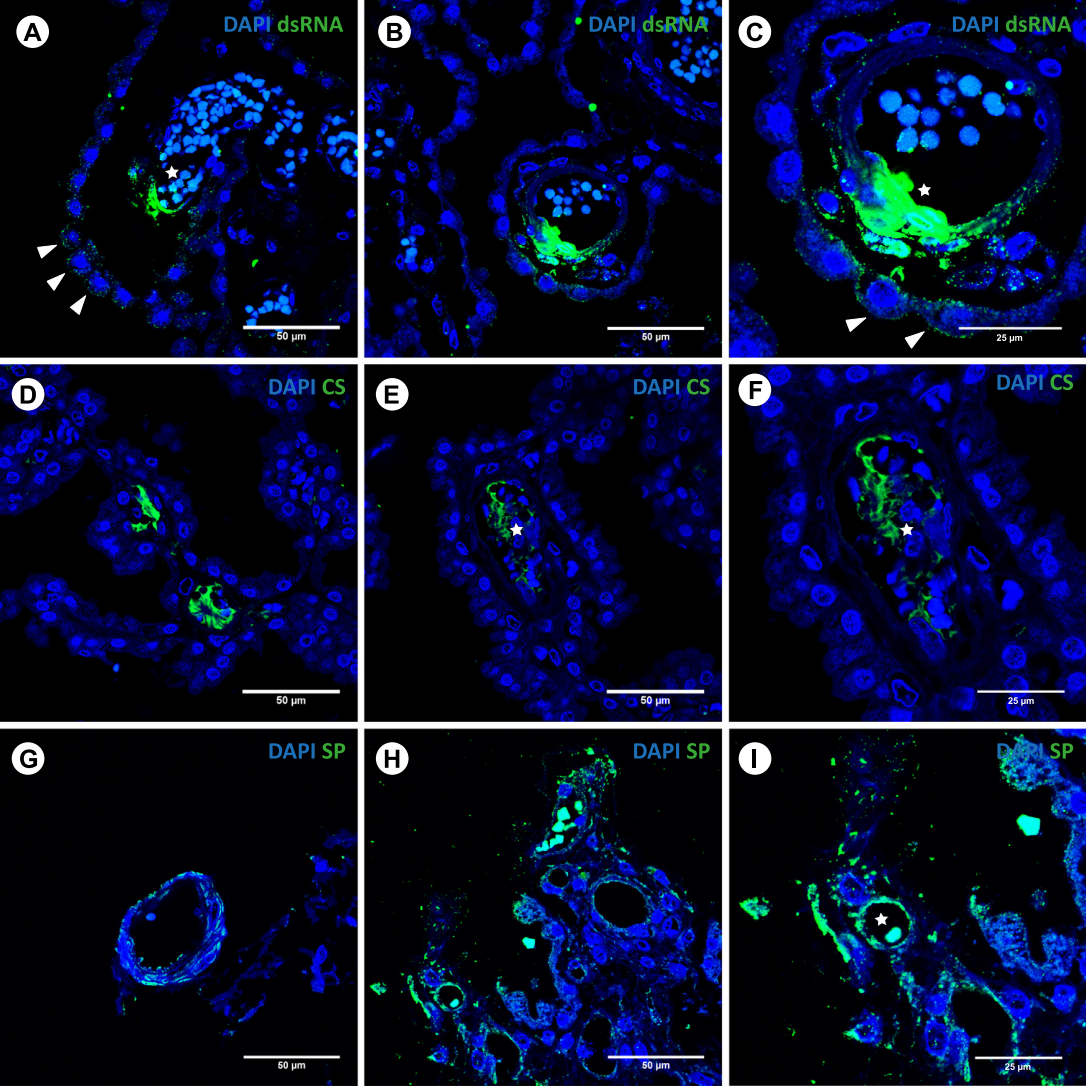
**Photomicrographs of immunostaining for SARS-CoV-2 in choroid plexus endothelium**. dsRNA (A-C), CS (D-F) and spike protein (G-I) stains in green and nuclear staining in blue (DAPI). SARS-CoV-2 infection of capillary lumina and vessels (stars) and adjacent cells (arrowheads). Scale bars: 50 µm. Details in (C), (F) and (I); Scale bars: 25 µm.

**Figure 4:**
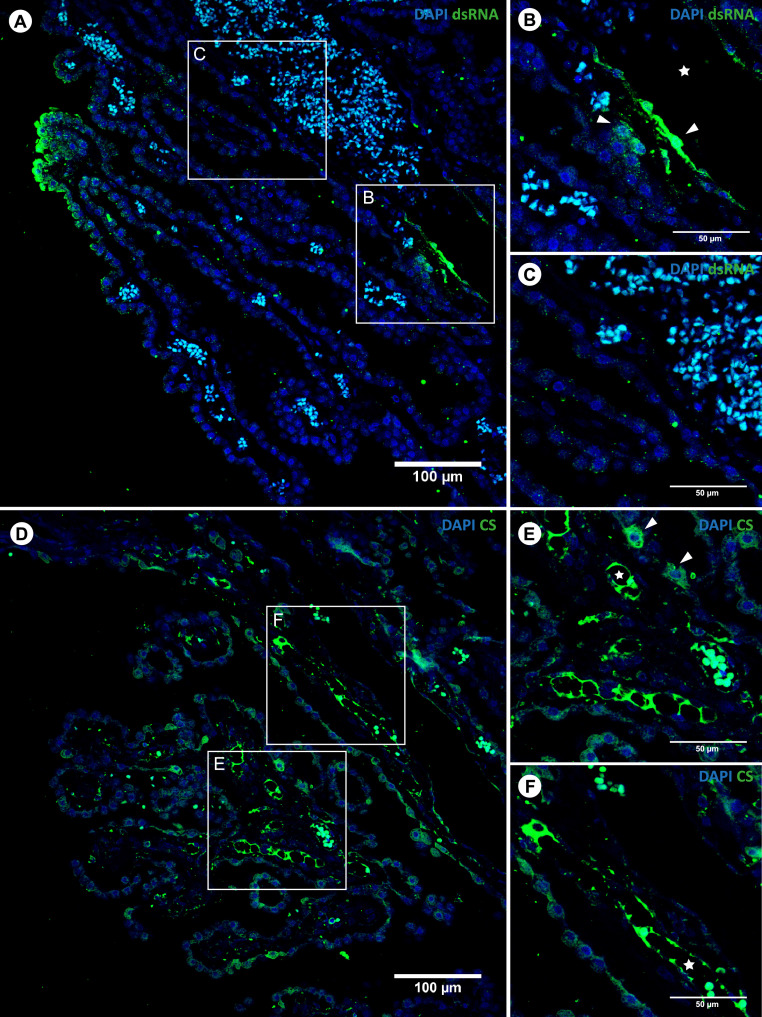
**Photomicrographs of immunostaining for dsRNA (A-C) and CS (D-F) in the choroid plexus endothelium.** Lower magnification images (**A** and **D**) showing SARS-CoV-2 infection in vessels and capillaries. Scale bars: 100 µm. Details in **B, C, E** and **F**. Scale bars: 50 µm. Note the presence of viral staining at the lumina (star) and nearby infected cells (arrowheads).

We found that SARS-CoV-2 infection was restricted at the lumina of ChP capillaries and medium-sized blood vessels, which was detected by immunostaining for double-stranded RNA (dsRNA) (Figure 3A-C), convalescent serum of a patient recovered from COVID-19 (CS) (Figure 3D-F), and SP (Figure 3G-I). In some vessels of the ChP, the entire wall was infected by SARS-CoV-2 (Figure 4E, F), whereas in others, clusters of cells adjacent to the infected endothelium had stronger immunoreactivity to SARS-CoV-2 than those in the adventitia (Figure 3A and C, Figure 4B, C and E).

## 4 Discussion

We report the *post-mortem* histopathological findings of a 14-month-old child who died of COVID-19. We observed a severe pneumonitis with massive cerebral atrophy and oedema and morphological features of hypoxic encephalopathy – a comorbidity that might have contributed to the poor outcome of COVID-19 – secondary to prolonged seizures and respiratory failure. We also demonstrated intense SARS-CoV-2 infections of the ChP epithelium and endothelium, some ependymal cells, and, to a lesser extent, the cerebral cortex.

The cause of death was a severe pneumonitis with diffuse respiratory bronchiolar and alveolar damage, together with massive ischemic necrosis of the pancreas secondary to a large vessel thrombosis, both previously reported in patients infected with SARS-CoV-2 [19,20]. In addition, the child presented multiple thromboses in several organs. *Post-mortem* studies in COVID-19 patients have shown microangiopathy and microthrombosis in major organs including the lungs [19,21]. Another hallmark of severe COVID-19 is the overproduction of proinflammatory cytokines which leads to systemic inflammation [22] and thrombosis [23,24]. We observed leukocyte infiltration, hyaline membranes, plugs of plasma proteins and cellular debris in the pulmonary parenchyma; and lymphoid depletion was also detected in lymphoid tissues, as previously reported [19].

The child's brain presented severe cortical neuronal loss, macrophage activation, and reactive gliosis, which are expected in encephalopathies. The encephalopathy reported here might have been related to hypoxia, secondary to both prolonged seizures and severe pneumonitis. A putative pre-existing condition (not diagnosed in life) contributing to this clinical status cannot be ruled out. Although we cannot determine the relative contribution of SARS-CoV-2 infection to our patient's encephalopathy, a recent study reported similar neurological complications of COVID-19 in previously healthy children, with or without co-infections [25]. In one fatal case, a child without comorbidities developed a marked choroid plexitis and fulminant CNS tuberculosis. The authors suggested that the breakdown of the blood-CSF barrier caused by SARS-CoV-2 infection of the ChP may have facilitated the entry of *Mycobacterium tuberculosis* into the CSF, resulting in a tuberculous superinfection of the CNS. Therefore, it is possible that our patient's SARS-CoV-2 infection substantially exacerbated a previously undiagnosed encephalopathy caused by a pre-existing condition.

Neurons and glial cells exhibit negligible surface expression levels of the ACE2 protein, the receptor and gateway for SARS-CoV-2 cellular entry. However, the ChP features the highest ACE2 expression level in the CNS [26,27]. Indeed, we found substantial SARS-CoV-2 immunoreactivity in the ChP and, to a lesser extent, in ependymal cells; and also detected the *N2* viral gene in the ChP. These findings are in accordance with previous *in vitro* studies showing SARS-CoV-2 infection in human ChP organoids [28,29]. The authors demonstrated that even though SARS-CoV-2 has minimal tropism for neurons and glial cells, it promotes breakdown of the brain-CSF barrier [29].

ChP has a single epithelial layer attached by tight junctions that establishes the blood-CSF barrier [30], [31], [32] SARS-CoV-2 inclusion bodies in endothelial cells with accompanying endotheliitis have been observed in patients with severe COVID-19 [33]. Given the essential role of the endothelium in vascular homeostasis, SARS-CoV-2-induced dysfunction may provoke thromboinflammation, resulting in vasculopathy [34]. Despite increasing evidence of COVID-19-related stroke in adults [35] and children [36], vasculitis was not observed in our patient's brain. However, we detected SARS-CoV-2 infection in ChP vasculature, consistent with a study that described viral-like particles in brain capillary endothelium, suggesting hematogenous dissemination as the most likely route to the CNS [36]. Therefore, SARS-CoV-2 infection of the ChP observed in this study could have disrupted the blood-CSF barrier, thus enabling neuroinvasion. In support of this hypothesis, SARS-CoV-2 RNA was detected in the CSF of a patient with COVID-19-related encephalitis [5].

## 5 Conclusions

Here we report multisystemic histopathological alterations caused by SARS-CoV-2 in a child, focusing on neuroinvasion and encephalopathy. Differences between the clinical phenotypes of COVID-19 in children and adults are still unclear; however, cases in both age groups seem to share critical hallmarks, including inflammation, thrombosis, and secondary tissue ischemia [22,^24^,^37^,38]. Despite the rarity of life-threatening paediatric COVID-19 cases, fatalities have been described in previously healthy children who developed severe encephalopathy, acute fulminant cerebral oedema, and ischemic or haemorrhagic stroke [36]. Adverse outcomes of COVID-19 in children such as death or chronic sequelae may become important public health issues in the future [39]. The severe encephalopathy reported here could have resulted from recurrent seizures due to an undiagnosed aetiology. Despite the putative prior encephalopathy, we cannot ignore its possible exacerbation due to SARS-CoV-2 infection. This report elucidates many aspects of paediatric SARS-CoV-2 infection that may contribute to the search for clinical and pharmacological strategies to counter COVID-19. Further studies may inform the clinical management of COVID-19 in both healthy and chronically ill children.

## Contributors

ICG, FCR and SR conceptualized the study. FCR performed the autopsy and full histological analysis. LC performed the neuropathological analysis. ICG performed material preparation for histological procedures and immunofluorescent staining. ICG, KK and CSGP performed confocal images analysis. MAM performed RNA isolation and RT-qPCR for viral detection. KK and LC prepared the figure panels. The first draft of the manuscript was written by KK, JTO and CSGP. All authors discussed the results and contributed to the final version of the manuscript. All authors read and approved the final manuscript. LC and SR coordinated the study.

## Declaration of interests

The authors declare no competing interests.

## Data sharing

The data generated and analyzed during the current study are available from the corresponding author on reasonable request.

## Appendix Supplementary materials

Image, application 1

Image, application 2

Image, application 3

Image, application 4

Image, application 5

Image, application 6
