# Supplementary figures and images for: SARS-CoV-2 infection of the central nervous system in a 14-month-old child: A case report of a complete autopsy
Source: Lancet Reg Health Am. 2021 Aug 28;2:100046. doi: 10.1016/j.lana.2021.100046 (PMC8397543; doi:10.1016/j.lana.2021.100046)

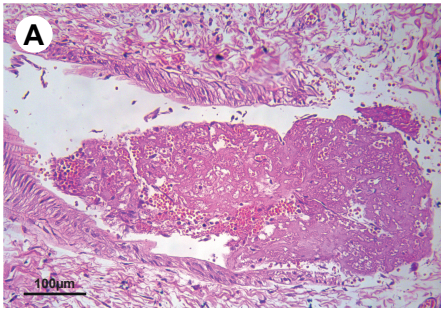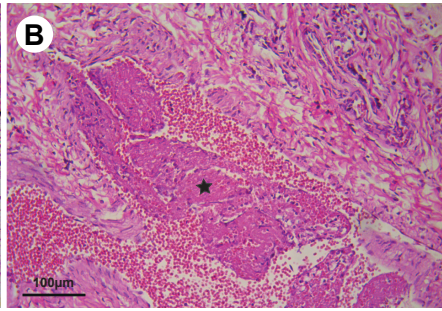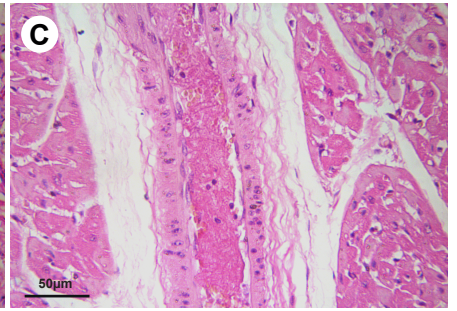

Supplement: Supplementary file 2 [file mmc2.pdf]

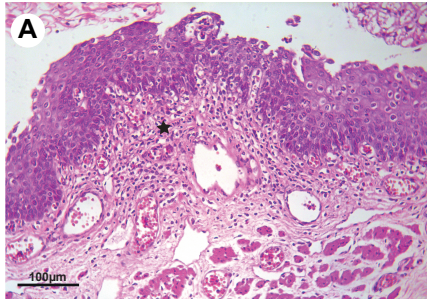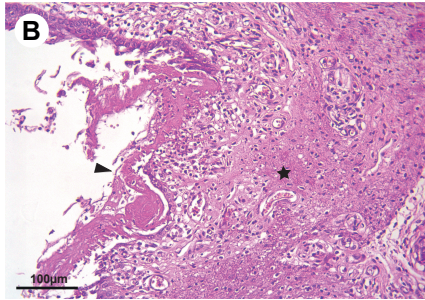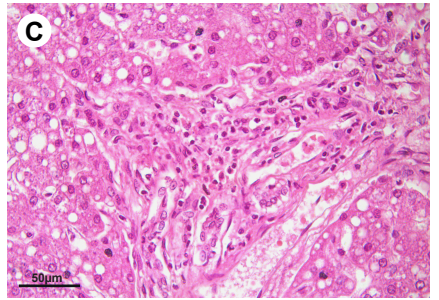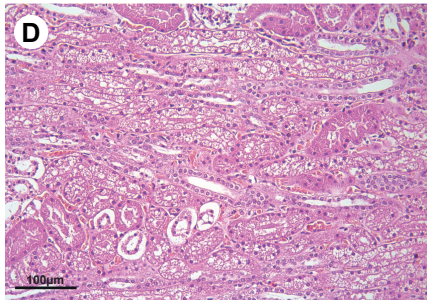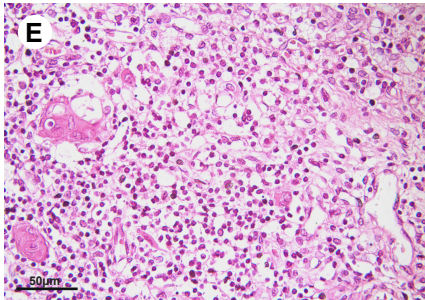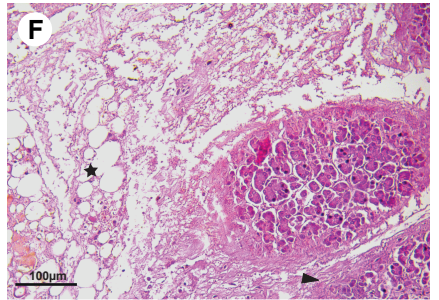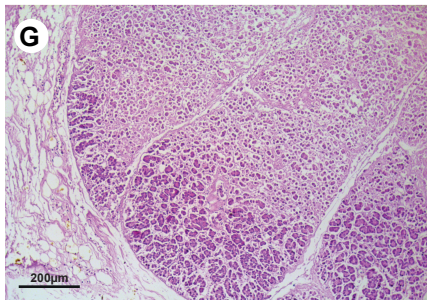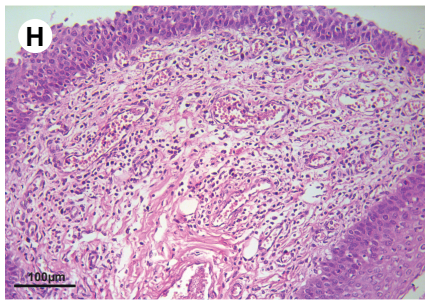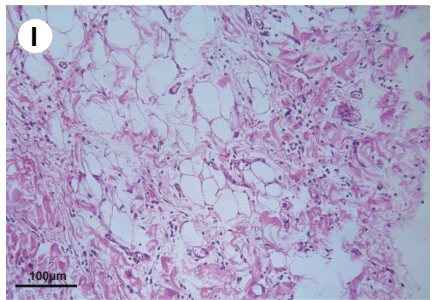

Supplement: Supplementary file 3 [file mmc3.pdf]

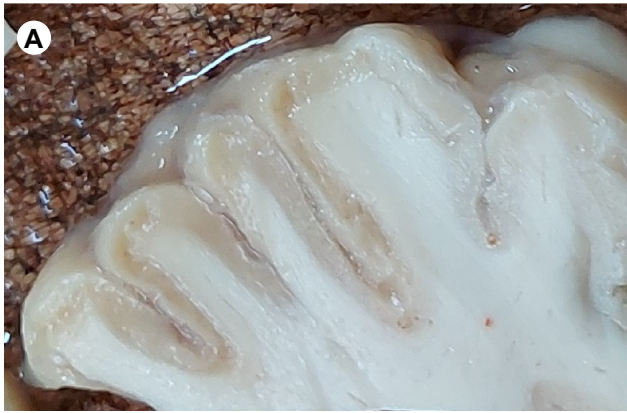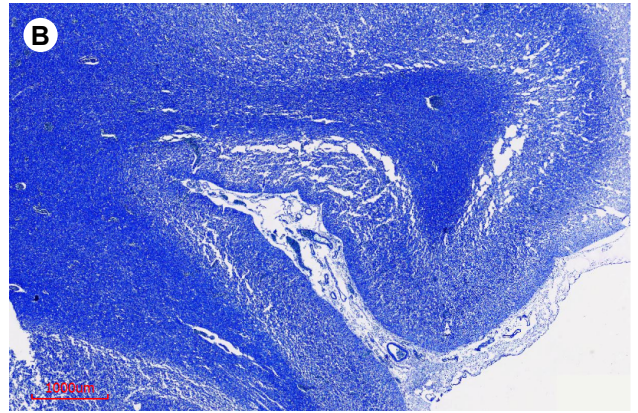

Supplement: Supplementary file 4 [file mmc4.pdf]

COVID-19

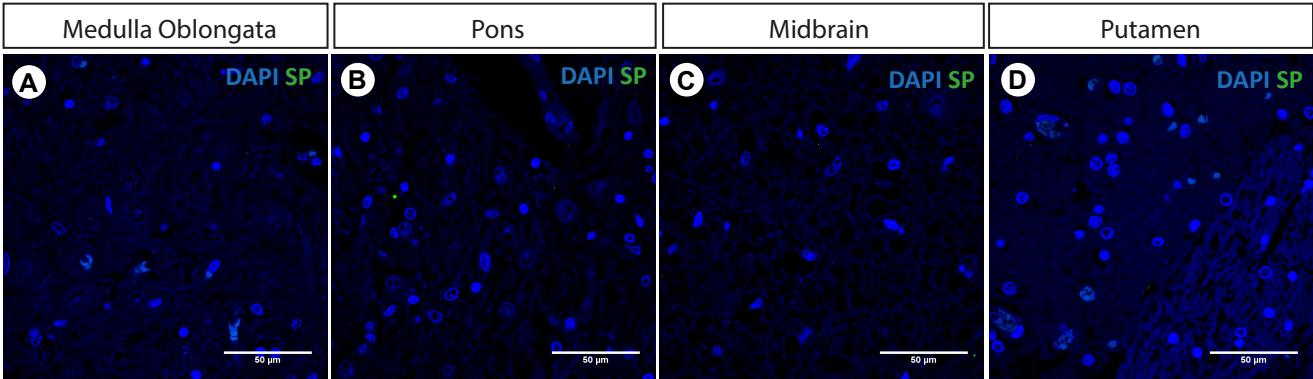

COVID-19 - Secondary antibody control

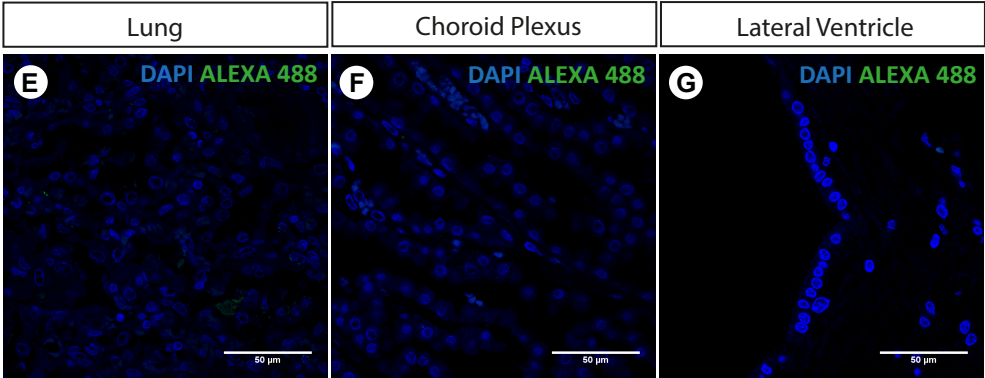

Non-COVID-19

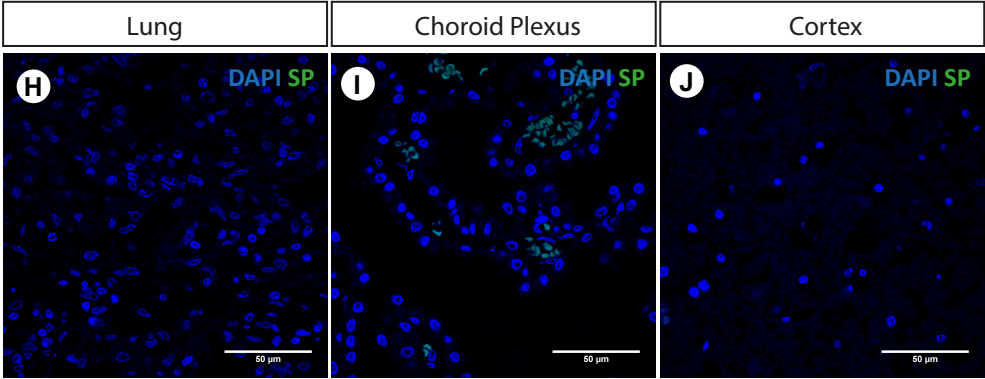

Supplement: Supplementary file 5 [file mmc5.pdf]

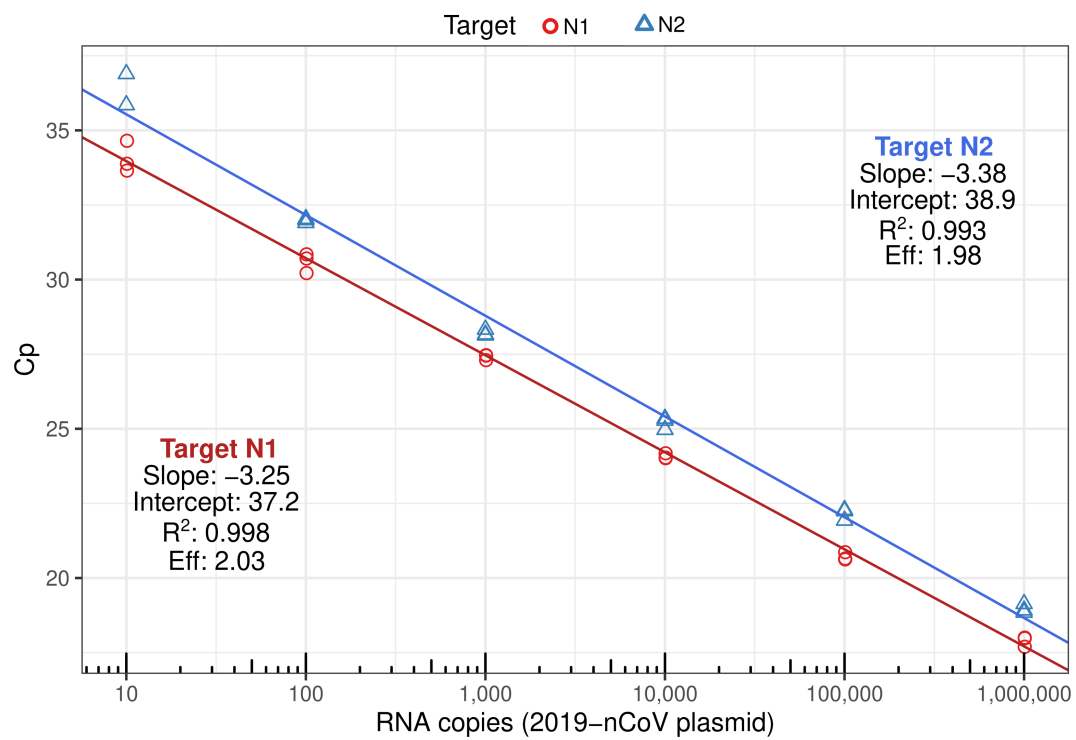

Supplement: Supplementary file 6 [file mmc6.pdf]
