# Supplementary material for: SARS-CoV-2 infection of the central nervous system in a 14-month-old child: A case report of a complete autopsy
Source: Lancet Reg Health Am. 2021 Aug 28;2:100046. doi: 10.1016/j.lana.2021.100046 (PMC8397543; doi:10.1016/j.lana.2021.100046)
Supplement: Supplementary file 1 [file mmc1.pdf]

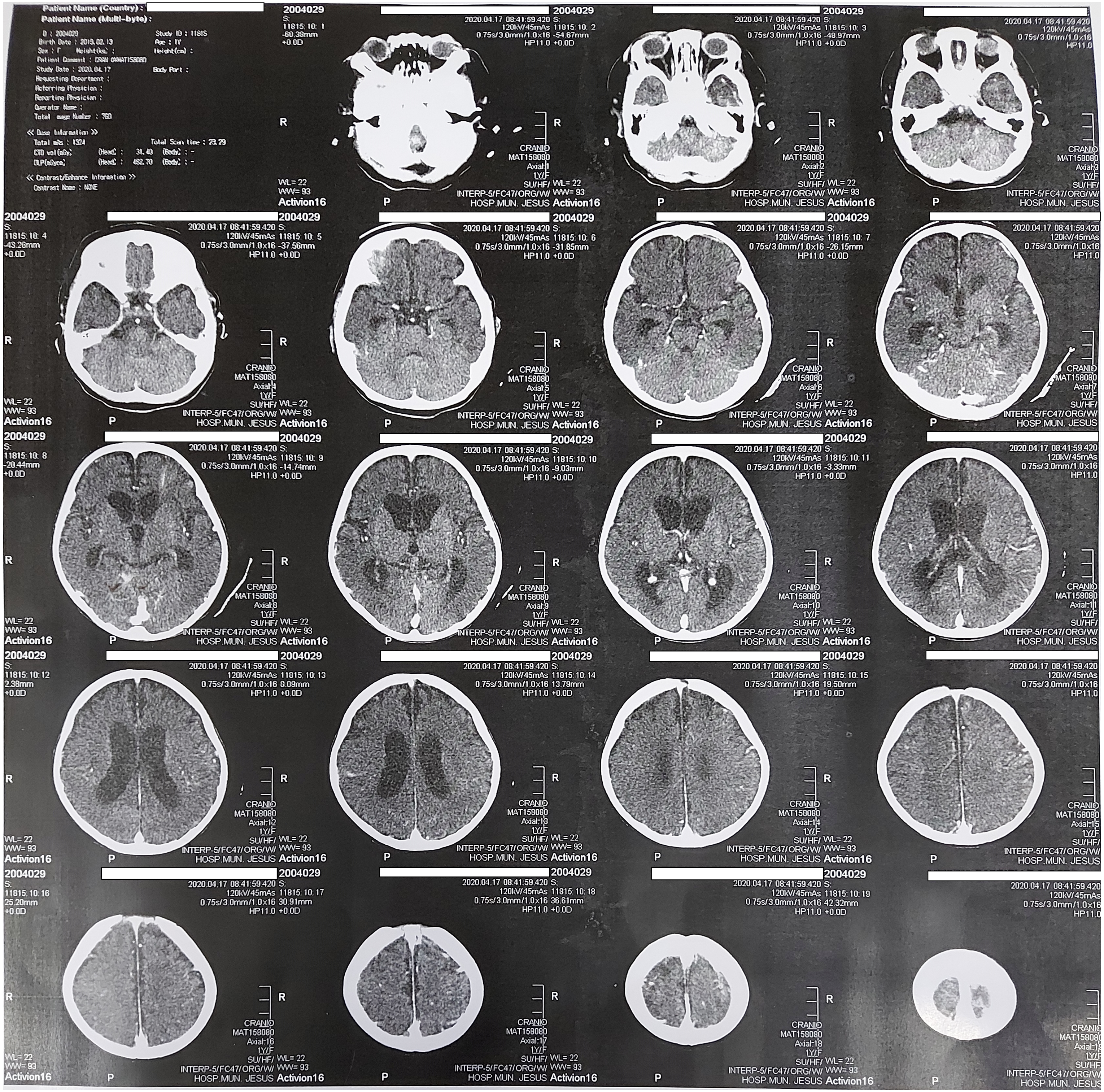
Patient Name (Country) : [redacted]
Patient Name (Multi-byte) :
D : 2004029
Study ID : 11815
Birth Date : 2019.02.13
Age : 1Y
Sex : F
Weight(kg) :
Height(cm) :
Patient Comment : CRAN 0MAT158080
Study Date : 2020.04.17
Body Part :
Requesting Department :
Referring Physician :
Reporting Physician :
Operator Name :
Total Image Number : 760
« Dose Information »
Total mAs : 1324
Total Scan time : 23.29
CTD vol(mGy) (Head) : 31.40 (Body) : -
DLP(mGycm) (Head) : 452.70 (Body) : -
« Contrast/Enhance Information »
Contrast Name : NONE
2004029
2020.04.17 08:41:59.420
120kV/45mAs
0.75s/3.0mm/1.0x16
HP11.0
S:
11815: 10: 1
-60.38mm
+0.0D
R
P
CRANIO
MAT158080
1Y/F
SU/HF/
INTERP-5/FC47/ORG/W/
HOSP.MUN. JESUS
WL= 22
WW= 93
Activion16
